# 3D QSAR, pharmacophore and molecular docking studies of known inhibitors and designing of novel inhibitors for M18 aspartyl aminopeptidase of *Plasmodium falciparum*

**DOI:** 10.1186/s12900-016-0063-7

**Published:** 2016-08-17

**Authors:** Madhulata Kumari, Subhash Chandra, Neeraj Tiwari, Naidu Subbarao

**Affiliations:** 1 Department of Information Technology, Kumaun University, SSJ Campus, Almora, Uttarakhand 263601 India; 2 Department of Botany, Kumaun University, SSJ Campus, Almora, Uttarakhand 263601 India; 3 Department of Statistics, Kumaun University, SSJ Campus, Almora, Uttarakhand 263601 India; 4 School of Computational and Integrative Sciences, Jawaharlal Nehru University, New Delhi, 110067 India

**Keywords:** *Plasmodium falciparum*, M18 aspartyl aminopeptidase, 3D QSAR, PLSR, PCR, kNN-MFA, Molecular docking, HTVS, Pharmacophore modeling

## Abstract

**Background:**

The *Plasmodium falciparum* M18 Aspartyl Aminopeptidase (*Pf*M18AAP) is only aspartyl aminopeptidase which is found in the genome of *P. falciparum* and is essential for its survival. The *Pf*M18AAP enzyme performs various functions in the parasite and the erythrocytic host such as hemoglobin digestion, erythrocyte invasion, parasite growth and parasite escape from the host cell. It is a valid target to develop antimalarial drugs. In the present work, we employed 3D QSAR modeling, pharmacophore modeling, and molecular docking to identify novel potent inhibitors that bind with M18AAP of *P. falciparum*.

**Results:**

The PLSR QSAR model showed highest value for correlation coefficient r^2^ (88 %) and predictive correlation coefficient (pred_r2) =0.6101 for external test set among all QSAR models. The pharmacophore modeling identified DHRR (one hydrogen donor, one hydrophobic group, and two aromatic rings) as an essential feature of *Pf*M18AAP inhibitors. The combined approach of 3D QSAR, pharmacophore, and structure-based molecular docking yielded 10 novel *Pf*M18AAP inhibitors from ChEMBL antimalarial library, 2 novel inhibitors from each derivative of quinine, chloroquine, 8-aminoquinoline and 10 novel inhibitors from WHO antimalarial drugs. Additionally, high throughput virtual screening identified top 10 compounds as antimalarial leads showing G-scores -12.50 to -10.45 (in kcal/mol), compared with control compounds(G-scores -7.80 to -4.70) which are known antimalarial M18AAP inhibitors (AID743024). This result indicates these novel compounds have the best binding affinity for *Pf*M18AAP.

**Conclusion:**

The 3D QSAR models of *Pf*M18AAP inhibitors provided useful information about the structural characteristics of inhibitors which are contributors of the inhibitory potency. Interestingly, In this studies, we extrapolate that the derivatives of quinine, chloroquine, and 8-aminoquinoline, for which there is no specific target has been identified till date, might show the antimalarial effect by interacting with *Pf*M18AAP.

**Electronic supplementary material:**

The online version of this article (doi:10.1186/s12900-016-0063-7) contains supplementary material, which is available to authorized users.

## Background

Malaria, a mosquito-borne disease, kills roughly 627000 people every year, mostly infants in Africa. It affects about 198 million patients annually (World Health Organization, 2013, http://www.who.int/malaria/media/en/). It is caused by parasites which are clubbed under genus *Plasmodium*. Among them, *P. falciparum* is encountered most commonly and is deadliest [1]. Though there are myriad drugs to treat the menace but the increasing instances of resistance against antimalarial drugs are becoming a deepening concern day by day. In recent years, several cases of resistance have been detected across the globe against artemisinin drugs [2]. This underscores the need to discover resilient drugs to combat malaria in future. Therefore, in this effort, several molecular drug targets have been identified to develop new drug candidates. An important drug target is M18 aspartyl aminopeptidase (M18AAP) which is expressed in the cytoplasm of *P. falciparum* by a single copy of *Pf*M18AAP gene. M18AAP interacts with the human erythrocyte membrane protein Spectrin and other proteins during disease kicking off erythrocytic life cycle, and it is essential for the survival of this parasite in Blood cells. It has been reported that the malaria parasites mutated with M18AAP enzyme are not able to survive, which proves that this plays a critical role in the survival of *P. falciparum* and could serve as an important molecular target to develop potential therapeutic agents to control malaria infection [3]. In modern times, virtual screening methods like QSAR, pharmacophore modeling, molecular docking have been proved a valuable tool for rapid discovery of novel drug candidates, e.g., the discovery of O-Acetyl-L-Serine Sulfhydrylase of *Entamoeba histolytica* inhibitors, acetylcholinesterase inhibitors, and antagonists Acetophenazine, fluphenazine and periciazine against Human androgen receptor [4–6]. In the drug development, the study of Quantitative structure-activity relationships (QSAR) plays an important role to analyze the properties of drugs. QSAR is a mathematical model that relates chemical descriptors of compounds to their quantity showing specific biological or chemical activity [7]. The molecular descriptors for the compounds are calculated and used to derive QSAR Model [8]. In the present study, the known bioactive dataset was used to build 3D QSAR models using partial least square regression (PLSR) [9], principal component regression (PCR) [10, 11] and k-nearest neighbor-molecular field analysis (kNN-MFA) methods [12]. After that, pharmacophore mapping was performed to identify the binding modes and structural features of the ligands and followed by molecular docking. The generated models provided a valuable reference which could be applied in the designing of pharmaceuticals with improved antimalarial activity. In the end, virtual screening of antimalarial compounds from ChEMBL Bioassay, and other dataset were also carried out to identify novel potential inhibitors which could be better as compared to the known inhibitors of *Pf*M18AAP.

## Methods

### Dataset of experimental *Pf*M18AAP inhibitors

A dataset of 32 compounds known as inhibitors of *Pf*M18AAP was extracted from National Center for Biotechnology Information PubChem bioassay (AID 743024) (https://pubchem.ncbi.nlm.nih.gov/assay/assay.cgi?aid=743024). Another high throughput screened dataset of 3502 known bioactive inhibitors of *Pf*M18AAP was extracted from AID 1822 used for docking studies against *Pf*M18AAP (http://pubchem.ncbi.nlm.nih.gov/assay/assay.cgi?aid=1822). A library of 153,873 compounds was obtained from the ChEMBL antimalarial database used for finding novel inhibitors against *Pf*M18AAP metalloproteinase [https://www.ebi.ac.uk/chembl/]. Additionally, 27 antimalarial drugs described by WHO, 32 analogous of quinine compounds(QN) (AID 660170), 24 analogous of chloroquine (CQ) (AID 404780), and 17 analogous of 8-aminoquinoline(8-AmQN) (AID 554037) were also extracted for molecular docking, 3D QSAR model and pharmacophore similarity search. 2D structures were converted to 3D structures using Corina 2.64v [13] and open babel [14].

### Molecular descriptors

The molecular descriptors were calculated by VLifeMDS version 4.3 using Gasteiger-Marsili charge [15, 16]. The *Pf*M18AAP inhibitors compounds along with their activity pIC50 values were given as input for force field calculation. The steric and electrostatic interaction energies are computed using a methyl probe of charge +1.

### Development of 3D QSAR models

The biological activity (pIC50) of inhibitors was selected as dependent variables and descriptors as independent variables. The 60 % data for the training set and 40 % for test set were manually selected. The unicolumn statistics were calculated to validate training and test sets. The 3D QSAR models were built using PLSR, PCR, and kNN-MFA by stepwise forward-backward method [17].

### 3D QSAR Model validation

#### Internal validation

To perform internal validation (cross validation), a compound is eliminated from the training set and then its biological activity is predicted to validate model accuracy. This step is repeated until the biological activity of every compound in the training set is predicted once. The cross-validated coefficient, q^2^ is calculated using the given Eq. (1):

1 $$ {q}^2=1-\frac{{\displaystyle \sum }{\left({y}_i-{\widehat{y}}_i\right)}^2}{{\displaystyle \sum }{\left({y}_i-{y}_{means}\right)}^2} $$

Where, y_i_ and ${\hat{y}}_i$ are the actual and predicted activities of the i^th^ molecule in the training set respectively, and y_means_ is the average activity of all the molecules in the training set [18, 19].

#### External validation

External validation (pred_r^2^) is carried out by calculating predicted correlation coefficient (pred_r^2^) value using following Eq. (2):

2 $$ pred\_{r}^2=1-\frac{{\displaystyle \sum }{\left({y}_i-{\widehat{y}}_i\right)}^2}{{\displaystyle \sum }{\left({y}_i-{y}_{means}\right)}^2} $$

Where, y_i_ and ${\hat{y}}_i$ are the actual and predicted activities of the i^th^ molecule in the test set, respectively, and y_means_ is the average activity of all the molecules in the training set.

A Z-score value is calculated by the following Eq. (3):

3 $$ Zscore=\frac{\left(h-\mu \right)}{\sigma } $$

Where, h is the q^2^ value calculated for the actual dataset, μ is the average q^2^and σ is the standard deviation calculated for various models built on different random datasets [20].

F-test is Fisher value which indicates statistical significance, a value greater than 30 is considered good, which gives an idea of the chances of failure of the model. On the other hand, q^2^_se is the standard deviation of cross validated prediction and r^2^_se is standard deviation is a measure of the absolute quality of a model.

### Pharmacophore modeling

The pharmacophore model was built using the Phase module of Schrodinger maestro [21]. The same set of inhibitors of *Pf*M18AAP was subjected to LigPrep module which produces high-quality, all-atom 3D structures with correct chirality. Some pharmacophore hypotheses were generated along with their respective set of aligned conformations. These hypotheses were generated by a systematic variation of many sites and a number of matching active compounds. These selected features were used to build a series of pharmacophore hypotheses by selecting find the common pharmacophore option in phase. The common pharmacophore hypotheses were analyzed using the survival score to yield the best alignment of the active ligands using a maximum overall root mean square deviation (RMSD) value of 2 Å for distance tolerance. Finally, several pharmacophore hypotheses were generated along with their respective set of aligned conformations. All pharmacophore hypotheses were scored for active survival, inactive survival, site, vector, volume, the number of matches, selectivity, energy, active, and inactive terms. Survival score secured by each hypothesis is the measure of the quality of alignment for a particular hypothesis [22].

### Docking and scoring

#### Molecular docking

To understand the nature of the interaction of inhibitors described above [23] with *Pf*M18AAP, molecular docking was performed using GOLD v5.2 (Genetic Optimization for Ligand Docking) [24] and GLIDE module of Schrödinger using [21] against the *Pf*M18AAP. The crystal structure of *Pf*M18AAP (4EME) was obtained from protein data bank (www.rcsb.org/pdb/explore/explore.do?structureId=4eme). Since *Pf*M18AAP requires cofactors for enzymatic activity, Zn was retained during docking analysis [25]. In GOLD docking, the 10 best docked complexes were ranked based on their GOLD fitness score. In GLIDE docking, the top 10 compounds were selected based on G-score. The binding affinity of docked complex was calculated using X-Score v1.2.1 [26]. Protein-ligand interaction was analyzed by using Pymol version 1.1r. www.pymol.org/ and LigPlot + v1.4.5 [27].

### Screening of PfM18AAP inhibitors

In this work, High Throughput Virtual Screening (HTVS) used Glide module of the Schrodinger software suite [21]. The ligand libraries were first prepared by adding hydrogen and generating conformations through the LigPrep module. This LigPrep module generated tautomer with the OPLS2005 force field, the total no. of 411,766 output structures were obtained. Then grid on the protein active site was generated. Firstly, HTVS for every ligand library was done and the top 1000 ranked compounds from every library were subjected to Extra-Precision (XP) screening. In both the cases, the structures were flexibly docked on the protein structure. The non-planar conformations were penalized. Structures were having more than 200 atoms or more than 35 rotatable bonds were not docked. Also, the Van Der Waal’s radius scaling factor was set to 0.8, and the partial charge cutoff was set to 0.15. From these 1000 compounds, the top 10 compounds from every library were extracted as target-bound complexes. These complexes were re-scored, and their binding affinity was calculated using X-score software.

## Results

### 3D QSAR modeling using PLSR Method

A dataset known as inhibitors of *Pf*M18AAP (AID: 743024) was used for the unicolumn statistics analysis, which showed that the training and test sets were suitable for 3D QSAR model development. The test set is interpolative i.e. derived within the min-max range of the training set. The unicolumn statistics scores were shown in Table 1. The PLSR model demonstrated that descriptors S_356, S_660, E_996, and S_270 are important features to inhibit the activity of *Pf*M18AAP, which represent steric and electrostatic field energy of interactions. The statistical parameters calculated for developed 3D QSAR model for PLSR shown in Table 2. The number suffixed with descriptors represents its position on the 3D spatial grid.

**Table 1:** Unicolumn statistics for training and test set

DataSet	Column Name	Average	Maximum	Minimum	Standard Deviation	Sum
Training	pIC50	5.6527	6.7200	5.1020	0.4450	90.4430
Test	pIC50	5.6559	6.3400	4.9200	0.4849	62.2146

**Table 2:** The statistical parameters for PLSR, PCR and 3D-QSAR models

Dependent variable	ZScore r^2^	ZScore q^2^	BestRand r^2^	BestRand q^2^	Z-Score Pred r^2^	Best-Rand Pred r^2^
PLSR pIC50	5.96671	2.43240	0.46222	-0.23735	1.64037	0.44031
PCR pIC50	5.11408	2.20918	0.43798	0.09365	1.39477	0.21574

Equation 4 represents the PLSR 3D QSAR model:

4 $$ \mathrm{pIC}50 = \hbox{-} 0.0270\ \left(\mathrm{S}\_356\right) + 0.0182\left(\mathrm{S}\_660\right)\ \hbox{-}\ 0.0905\left(\mathrm{E}\_996\right)\ \hbox{-}\ 0.0125\left(\mathrm{S}\_270\right) + 6.1966 $$

### 3D QSAR modeling using PCR

The 3D QSAR Model was developed on the same datasets of molecules by PCR method, and several statistical parameters were calculated which are shown in Table 2. The number suffixed with descriptors represents its position on the 3D spatial grid. This model indicated that descriptors are significant for their biological activities.

Equation 5 represents PCR 3D QSAR model:

5 $$ \mathrm{pIC}50 = \hbox{-} 0.0321\left(\mathrm{S}\_356\right) + 0.0147\left(\mathrm{S}\_660\right)\ \hbox{-}\ 0.0886\left(\mathrm{E}\_996\right)\ \hbox{-}\ 0.0092\left(\mathrm{S}\_270\right) + 6.3423 $$

### 3D QSAR Modeling using (kNN-MFA)

The kNN-MFA model shown that the contributing descriptors E_862 (1.0026 1.1562), S_629 (-0.4639 -0.1045) and S_287 (-0.3372, -0.2663) which indicated that degree of amino group shows potent activity. The range at the lattice point E_862 (1.0026, 1.1562) which is positive that means substitution with more electron density could yield more active molecules.

### Pharmacophore-based screening of *Pf*M18AAP inhibitors

**Fig. 1:**
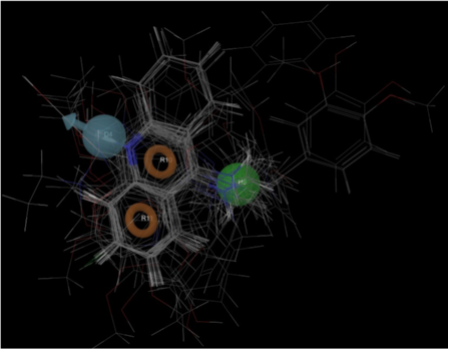
Diagram showing pharmacophore alignments of known *Pf*M18AAP inhibitors (AID 743024)

From the Phase Software, ten hypotheses (pharmacophore models) were generated having four features DHRR (one hydrogen bond donor (D), hydrophobic groups (H) and two aromatic rings (R)). These features were common to all of the 15 compounds of the assay. Common pharmacophore hypothesis is shown in Fig. 1. The best model was chosen based on the survival score and pharmacophore based QSAR. The final hypothesis, DHRR.31 model, was selected based on the survival score and pharmacophore based QSAR, which showed the best alignment of the active set along with the site score (0.79), vector score (0.949), and volume score (0.527), top 5 model is shown in Table 3.

**Table 3:** The statistical values of top 5 the pharmacophore hypotheses

ID	Survival	Survival inactive	Site	Vector	Volume	Selectivity	Matches	Energy	Activity	Inactive
DHRR.31	11.068	9.052	0.79	0.949	0.527	1.466	14	0.001	6.34	2.016
DHRR.27	11.068	9.052	0.79	0.949	0.527	1.466	14	0.001	6.34	2.016
DHRR.6	10.941	8.863	0.78	0.943	0.548	1.471	14	0.551	6.22	2.079
DHRR.15	10.941	8.863	0.78	0.943	0.548	1.471	14	0.551	6.22	2.079
DHRR.26	10.892	8.81	0.79	0.944	0.535	1.47	14	0	6.15	2.082

### Molecular docking

**Fig. 2:**
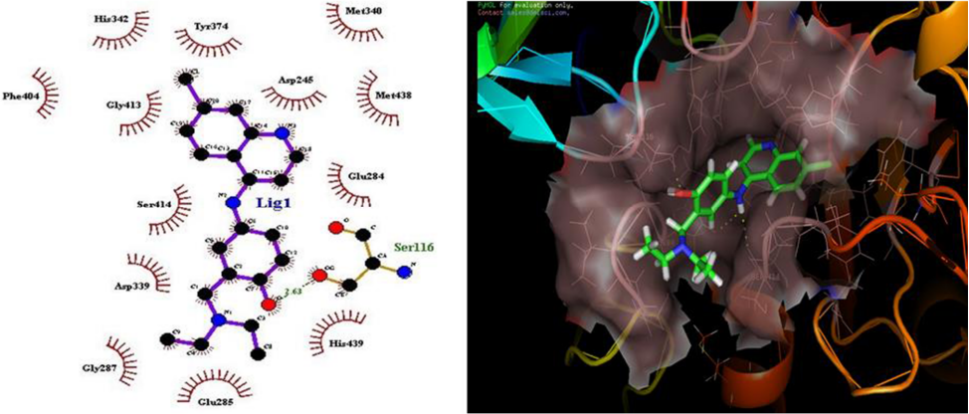
Docked Complex of *Pf*M18AAP with known ligand 4-[(7-chloroquinolin-4-yl) amino]-2-(diethylaminomethyl) phenol

The same data set used for QSAR and Pharmacophore modeling was subjected to the molecular docking analysis. The top 10 compounds showed GOLD fitness score from 60.62 to 39.81 and predicted binding energy from -6.43 to -7.38 kcal/mol (calculated using the X-Score) and G-score from -7.80 to -4.70 kcal/mol (Table 4). The Ligplot + analysis showed that Ser116 and His87 amino acids interact by h-bond interaction, with docked ligands. Since *Pf*M18AAP requires a cofactor for enzymatic activity, docking was performed along with cofactor bound with specific amino acids. A docked complex is depicted in Fig. 2. These results suggest that the novel *Pf*M18AAP inhibitors could be designed considering parameters of docking results leading to new potent drugs against malaria.

**Table 4:** Top scoring compounds screened using the selected pharmacophore hypothesis

Compound ID	G-Score (kcal/mol)	Align Score	Vector Score	Volume Score	Fitness	Predicted activity (pIC50)
CHEMBL588000	-10.33	1.4702	0.0537	0.3833	0.2119	5.72
CHEMBL587141	-10.12	0.8484	0.8644	0.4971	1.6545	5.83
CHEMBL529157	-9.81	1.7562	0.3816	0.3651	0.2833	5.85
CHEMBL528484	-9.79	1.5091	0.6425	0.3672	0.7521	5.86
CHEMBL532976	-9.52	1.2208	0.7452	0.2344	0.9623	6.07
CHEMBL2414638	-9.41	0.4596	0.9888	0.3135	1.9194	5.97
CHEMBL601831	-9.37	1.0285	0.6596	0.29897	1.1014	5.85
CHEMBL390368	-9.24	1.0146	0.9530	0.3788	1.4863	5.89
CHEMBL591216	-8.72	0.5189	0.6304	0.3387	1.5367	5.84
CHEMBL465847	-8.08	0.6220	0.7935	0.3477	1.6228	5.87

Molecular docking analysis was done on another dataset (AID1822:3502 molecules from PubChem Bioassay) known inhibitors of *Pf*M18AAP. The top 10 compounds showed G-score from -7.72 to -6.52 kcal/mol. The G-score indicated that these compounds (Table 5) might bind to *pf*M18AAP with good binding affinity. Further, predicted binding affinity calculated using X-score for best compounds was found to be in between from -9.54 to -6.51 kcal/mol (Table 5).

**Table 5:** Prediction of pIC50 Value of current antimalarial drugs described in the WHO

Compound ID	Generic Name	G-Score (kcal/mol)	Align Score	Vector Score	Volume Score	Fitness	Predicted activity (pIC50)
CHEMBL76	Chloroquine	-3.80	0.1086	0.9996	0.5197	2.4288	6.208
CHEMBL1535	Hydroxychloroquine	-4.53	0.1995	0.9973	0.3341	2.1652	6.207
CHEMBL303933	Piperaquine	-5.30	0.2720	0.9781	0.3049	2.0563	6.19
CHEMBL506	Primaquine	-5.86	0.4463	0.8889	0.4954	2.0124	6.192
CHEMBL2104009	Amquinate	-5.41	0.6142	0.9499	0.355	1.7931	6.205
CHEMBL416956	Mefloquine	-5.28	0.5712	0.7183	0.3248	1.5672	6.20
CHEMBL682	Amodiaquine	-4.48	0.6480	0.7838	0.2687	1.5126	6.385
CHEMBL36	Pyrimethamine	-5.07	0.9521	0.9257	0.3390	1.4712	6.207
CHEMBL339049	Tebuquine	-4.55	0.6093	0.7422	0.2286	1.4630	6.264
CHEMBL35228	Pyronaridine	-5.68	0.6975	0.8185	0.2050	1.4422	6.198

### HTVS based screening of *Pf*M18AAP inhibitors

**Fig. 3:**
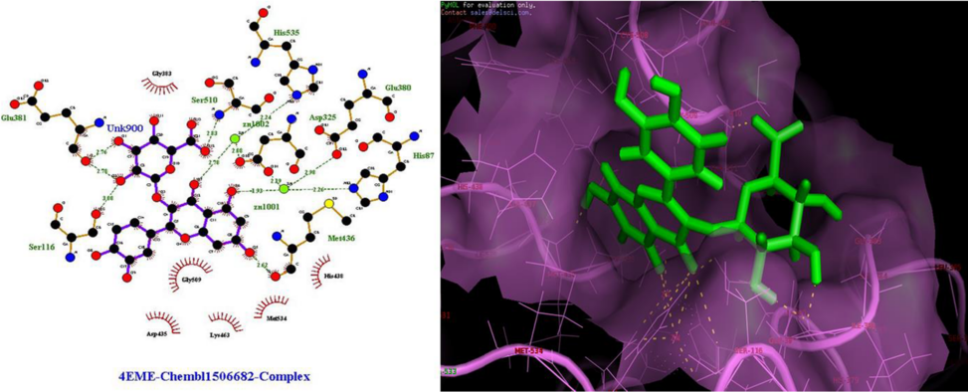
Ligplot diagram and docked Complex of *Pf*M18AAP with ligand ChEMBL Database Compound [2-(3,4-Dihydroxyphenyl)-5,7-dihydroxy-4-oxo-4H-chromen-3-yl hexopyranosiduronic acid]

ChEMBL antimalarial dataset (153873) was subjected to molecular docking. The top 10 compounds (after docking), based on their G-score are shown in Table 6. The glide score of these compounds varies from -12.50 to -10.45 kcal/mol. The G-score indicated that these compounds (Table 6) have a good binding affinity for *Pf*M18AAP enzyme. Figure 3 shows the docked complex of ligand CHEMBL1506682 (2-(3,4-Dihydroxyphenyl)-5,7-dihydroxy-4-oxo-4H-chromen-3-yl hexopyranosid-uronic acid) in the active site of the receptor with best G-score (-12.50 kcal/mol).To further validate *in silico*, predicted binding affinity of the best pose obtained from docking studies for each compound was calculated using X-score program was found to be in between -8.28 and -6.89 kcal/mol shown in Table 6.

**Table 6:** Top scoring of QN, CQ and 8 Amino-QN analogous screened using the selected pharmacophore hypothesis

IUPAC Name	G-Score (kcal/mol)	Align Score	Vector Score	Volume Score	Fitness	Predicted activity (pIC50)
(9S)-Cinchonan-9-ol	-4.18	0.8099	0.5259	0.4368	1.2878	5.521
(9S)-6′-Methoxycinchonan-9-ol	-5.47	1.0187	0.7020	0.2737	1.1268	5.85
N-(7-Chloro-4-quinolinyl)-N’-ethyl-1,4-butanediamine	-3.84	0.1101	0.9993	0.5	2.4075	5.98
1,4-Pentanediamine, N4-(7-chloro-4-quinolinyl)-N1,N1-diethyl-Chloroquine	-3.52	0.1053	0.9983	0.4755	2.3861	5.86
PrimaquineN^4^-(6-Méthoxy-8-quinoléinyl)-1,4-pentanediamine	-5.14	0.5014	0.9017	0.4005	1.8844	5.75
N^4^-{2,6-Diméthoxy-4-méthyl-5-[3-(trifluorométhyl)phénoxy]-8-quinoléinyl}-1,4-pentanediamine	-5.32	0.5274	0.9118	0.2755	1.7478	5.67

## Discussion

**Fig. 4:**
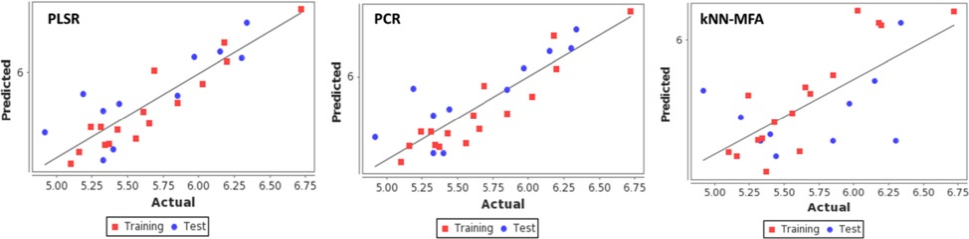
Scatter plots showing the correlation between actual versus predicted activities for training and test set molecules by using 3D QSAR model- PLSR, PCR, and kNN-MFA

**Fig. 5:**
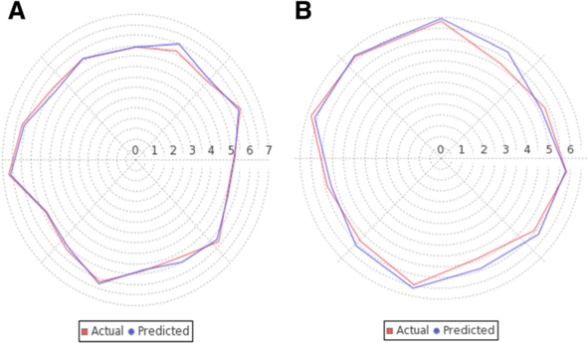
Radar plots showing the actual and predicted activities for **a** Training set **b** Test set molecules by using 3D QSAR PLSR model

**Fig. 6:**
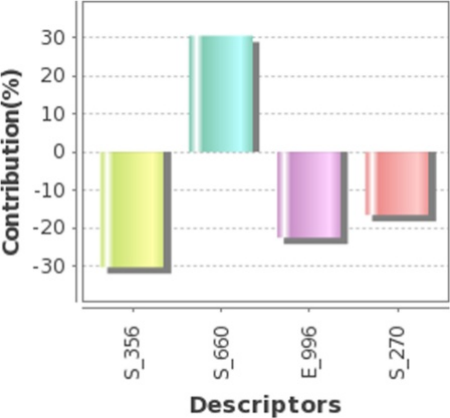
Plot of the percentage contribution of each descriptor in developed 3D QSAR PLSR model explaining variation in the activity

The best model was selected through the comparison between fitness plots (Fig. 4) and radar plots for training and test sets (Fig. 5 (a, b)). The linear graphical representation of fitness plots shows the observed and predicted activities of the data set. The radar plots show the training and the test sets separately by the red (actual activity) and blue (predicted activity) lines. The radar plot for training set represents a good r^2^ value because the two lines show a good overlap while for the test set a good overlap represents high pred_r^2^ value. The PLSR contribution plot for the descriptor is given in Fig. 6 which represents the contribution of various descriptors which are important for the inhibitory activity. In PLSR and PCR models, the negative value in electrostatic field descriptors indicates that negative electronic potential is required to increase antimalarial activity, and more electronegative groups are preferred in that position. Though positive value in kNN-MFA model shows that group that imparting positive electrostatic potential is favorable for antimalarial activity, so less electronegative group should prefer in that region. Similarly, negative values in steric descriptors indicate that negative steric potential is favorable for activity, and less lipophilic substitutions or bulky substituents group should be considered in that region, positive value of steric descriptors reveals that positive steric potential is favorable to increase antimalarial activity as in case of 4-[2-(quinolin-4-ylamino)ethyl] benzene-1,2-diol, and more bulky group is advised to prefer in that region. Comparison of statistical parameters of PLSR, PCR, and kNN-MFA, is shown in (Additional file 1) and the predicted pIC50 values in (Additional file 2).

In the present work, we performed screening of CHEMBL antimalarial library to search antimalarial compounds based on the pharmacophoric hypothesis DHRR.31, which resulted in 29,671 compounds. These compounds were subjected to glide docking against *Pf*M18AAP. The top 10 compounds were selected based on the fitness and G-score; predicted activities are shown in Table 7. Further we also carried out screening of 27 WHO antimalarial drugs which resulted in 14 molecules shown in Table 8. Moreover, 17 compounds of 8-aminoquinolines analogous, 24 compounds of CQ analogous and 32 compounds of 8 amino-QN analogous were subjected to screening resulting 17,19, and 22 *Pf*M18AAP inhibitors respectively (Table 8). The resultant top 2 compounds from each analogous were selected based on the fitness and G-score; predicted activities are shown in Table 9. The study found that WHO current antimalarial compound CHEMBL682 (Amodiaquine) has highest predicted value of pIC50 6.38 which is also present in the known dataset of *Pf*M18AAP with pIC50 value 6.72.

**Table 7:** Molecular Docking Results for known inhibitors (AID743024) against *Pf*M18AAP

IUPAC Name	Gold Score	G-Score (kcal/mol)	X-Score (kcal/mol)	H Bond	No. of Hydrophobic Interaction	No. of NB Interactions	pIC50 Value
4-[(7-chloroquinolin-4-yl)amino]-2-(diethylamino methyl)phenol	36.57	-5.35	-8.09	Ser116	13	33	6.72
7-chloro-N-[2-(3,4-dimethoxyphenyl)ethyl]quinolin-4-amine	35.17	-5.40	-7.48	-	11	60	6.18
N-[2-(3,4-dimethoxyphenyl)ethyl]-6-ethoxyquinolin-4-amine	33.65	-6.43	-7.08	His342	9	72	5.85
N-[2-(3,4-dimethoxyphenyl)ethyl]isoquinolin-4-amine	33.45	-4.97	-7.17	-	11	59	5.34
4-[2-[(7-chloroquinolin-4-yl)amino]ethyl]benzene-1,2-diol	32.56	-7.80	-7.38	Ser414	12	70	6.2
3-[2-(quinolin-4-ylamino)ethyl]benzene-1,2-diol	32.41	-5.67	-7.36	Glu284 Ser414	10	77	5.56
N-[2-(2-bromo-4,5-dimethoxyphenyl)ethyl]quinolin-4-amine	32.31	-4.85	-7.35	-	11	61	6.34
1-benzyl-N-[2-(3,4-dimethoxyphenyl)ethyl]piperidin-4-amine	32.11	-4.70	-7.10	Ser116	11	61	5.16
4-[2-(quinolin-4-ylamino)ethyl]benzene-1,2-diol	31.89	-5.25	-7.19	Glu284 Ser414	10	62	5.4
4-[3-(acridin-9-ylamino)propyl]benzene-1,2-diol	30.58	-5.65	-7.63	His87 Asp89	6	46	5.43

*H Bond* Hydrogen-Bond, *NB* Non Bonded

**Table 8:** Molecular Docking Results for known inhibitors (AID1822) against *Pf*M18AAP

S. No.	Chemical Substance ID	G-Score (kcal/mol)	X-Score (kcal/mol)	HBond	No. of Hydro-phobic Interactions	No. of NB Interactions	% Inhibition
C1	49644635	-7.72	-8.42	Gly509	8	68	32.65
C2	24707924	-7.71	-9.54	Ser116, Asp325, Met436, Lys463	8	76	75.26
C3	26665815	-7.48	-6.51	Ser116, Cys508	4	32	31.93
C4	50086555	-7.36	-7.66	Ser116, Glu380, His438, Ser510	7	35	55.6
C5	49647140	-7.143	-7.04	Ser116, Met436, His438, Lys463	7	29	55.21
C6	47195345	-7.11	-8.14	His438, Asp325, Glu380, His87, His535	7	37	28.24
C7	49644096	-7.07	-7.37	Asp325, Glu380, Ser510, His 535	4	39	37.43
C8	24779308	-6.88	-7.29	His 87,Asp325, Glu380,His535	6	38	37.29
C9	17504161	-6.57	-7.92	Ser116, Asp435, Met436, Lys463	9	32	53.68
C10	11532952	-6.52	-7.57	His438	9	36	36.43

**Table 9:**
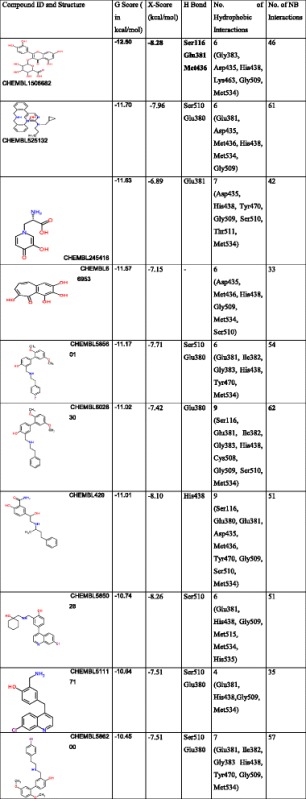
Top scoring 10 potential inhibitors from CHEMBL antimalarial Library against *Pf*M18AAP

We analyzed the types of interactions of each top ranked compound for known inhibitors (AID1822) against *Pf*M18AAP; 2D plots were generated using Ligplot + software and ligand-protein complex. The number of hydrogen bonded interactions, lipophilic interactions and the number of non-bonded interactions was counted and tabulated in Table 5. It is observed that overall all compounds from C1 to C10 have formed at least 1 (C1 and C10), mostly 4 (C3, C4, C7, C8, and C9), and at most 5 (C6) hydrogen bonds. The total number of lipophilic interactions for each compound varies in between 9 (for C9, C10) and 4 (for C3 and C7). Also, the total number of non-bonded interactions for each compound varies from 29 (for C5) to 76 (for C2). These observations suggest that the compounds C3, C4, C6, C7, C8, and C9 have better specificity as they have more hydrogen bonds and compounds C1, C2, C9, and C10 have good binding affinity due to a high number of hydrophobic contacts. The Compound C1 showed interaction with Glide score -7.72 kcal/mol. The docking poses analysis of C1shows one hydrogen bond (Gly509) interaction with amino acid residues of the protein. The next favorable interaction is shown by C2 with G-score of -7.71 kcal/mol and four hydrogen bond interactions with the active site residues Ser116, Asp325, Met436 and Lys463, 76 nonbonded interactions and inhibition (75.26 %) and eight hydrophobic interactions. The Compound C6 showed highest five hydrogen bond interaction (His438, Asp325, Glu380, His87, and His535). **Asp325** is found to be the most conserved residues, which is present in 6 out of 10 compounds and **Ser116** is found to be the most conserved residues, which is present in 5 out of 10 compounds. Hence, based on the Docking analysis against antimalarial *Pf*M18AAP inhibitors, we conclude that these compounds have a better affinity with *Pf*M18AAP enzyme, thus are novel potential candidate to develop drugs against malaria.

Further, we also analyzed the interactions of CHEMBL antimalarial library’s top ranked inhibitors against *Pf*M18AAP (Table 6). The highest X score of - 11.6 kcal/mol was obtained with the ligand (CHEMBL1506682) having three hydrogen bond (**Ser116, Glu381, and Met436**) interaction with amino acid residues of the protein. The total number of lipophilic interaction for each compound varies in between 9 (CHEMBL602830 and CHEMBL429) and 4 (for CHEMBL511171). This observation suggests that CHEMBL1506682 have better specificity and CHEMBL602830 have a good binding affinity. **Ser510** and **Glu38**0 are found to be the most conserved residues, which is present in 5 out of 10 compounds. Hence, based on the comparison between known bioactive antimalarial M18AAP inhibitors (as control) and top ten novel ChEMBL compounds, we conclude that these compounds could bind to *Pf*M18AAP with better affinity, thus are the potential candidate to develop drugs against malaria.

## Conclusions

The present study was aimed at generating the predictive 3D QSAR models capable of revealing the structural requirements for antimalarial inhibitors of *Pf*M18AAP. The comparison of the different statistical parameters of the three models suggests that PLSR model is best due to better internal validation q^2^_=_ 0.6128 and an external test of pred_r^2^_=_ 0.6101. Model 3 (kNN-MFA) also had a good internal validation showing q^2^_=_0.7641, but the external validation had a bad pred_r^2^_=_ 0.0366. Therefore both PLSR and PCR models show potential predictive ability as determined by testing the external test set. Thus, 3D QSAR modeling provided a better understanding of the structural requirements of antimalarial compounds, which could help design potent *Pf*M18AAP inhibitors. Also, pharmacophore mapping was applied to identify the binding modes and structural features of the ligands which are important for the biological activity of the inhibitors. The pharmacophore modeling showed that hypothesis DHRR.31 represented the best pharmacophore model for determining *Pf*M18AAP inhibitory activity. Results suggested that the proposed DHRR.31 model can be used to identify the new M18AAP inhibitor and to design a drug rationally for *p. falciparum* from the extensive 3D database of molecules. Further, HTVS using Glide resulted in several potent *Pf*M18AAP inhibitors from ChEMBL antimalarial data set of 153,873 compounds. These novel compounds having an excellent binding affinity with *Pf*M18AAP are better candidates to design the drug in future. Finally, the 3D QSAR model was deployed on different data set to prioritize *Pf*M18AAP inhibitors and predict new inhibitors. Thus, our study advocates the use of combined approaches of 3D QSAR, pharmacophore modeling, and molecular docking to search for novel potential inhibitors unique to *Pf*M18AAP, which is essential and validated drug target involved in performing various enzymatic functions such as hemoglobin digestion, erythrocyte invasion, and parasite growth in the host cell.

## Additional files

Additional file 1: The statistical parameters of 3D QSAR models of known bioactive Inhibitors (AID 743024) dataset of *Pf*M18AAP using PLSR, PCR and kNN-MFA methods. (DOCX 16 kb) Additional file 2: Comparison between different 3D QSAR models using PLS, PCR and KNN methods for predicting pIC50 values of train set and test set of known bioactive Inhibitors (AID 743024) of *Pf*M18AAP. (DOCX 18 kb)

## Abbreviations

3D QSAR: 3-dimensional quantitative structure activity relationship
*Pf*M18AAP: *Plasmodium falciparum* M18 Aspartyl Aminopeptidase
PLSR: Partial least square regression
PCR: Principal component regression
kNN-MFA: k-nearest neighbor-molecular field analysis
QN: Quinine
CQ: Chloroquine
8-AmQN: 8-aminoquinoline
HTVS: High Throughput Virtual Screening
*P. falciparum*: *Plasmodium falciparum*

## References

[1] Newton CR, Krishna S (1998). Severe falciparum malaria in children: current understanding of pathophysiology and supportive treatment. Pharmacol Ther.

[2] Basco LK, Le Bras J (1993). In vitro activity of artemisinin derivatives against African isolates and clones of Plasmodium falciparum. Am J Trop Med Hyg.

[3] Lauterbach SB, Coetzer TL (2008). The M18 aspartyl aminopeptidase of Plasmodium falciparum binds to human erythrocyte spectrin in vitro. Malar J.

[4] Nagpal I, Raj I, Subbarao N, Gourinath S (2012). Virtual screening, identification and in vitro testing of novel inhibitors of O-acetyl-L-serine sulfhydrylase of Entamoeba histolytica. PLoS One.

[5] Mizutani MY, Itai A (2004). Efficient method for high-throughput virtual screening based on flexible docking: discovery of novel acetylcholinesterase inhibitors. J Med Chem.

[6] Bisson WH, Cheltsov AV, Bruey-Sedano N, Lin B, Chen J, Goldberger N, May LT, Christopoulos A, Dalton JT, Sexton PM, Zhang XK, Abagyan R (2007). Discovery of antiandrogen activity of nonsteroidal scaffolds of marketed drugs. Proc Natl Acad Sci U S A.

[7] Esposito EX, Hopfinger AJ, Madura JD (2004). Methods for applying the quantitative structure-activity relationship paradigm. Methods Mol Biol.

[8] Xue L, Bajorath J (2000). Molecular descriptors in chemoinformatics, computational combinatorial chemistry, and virtual screening. Comb Chem High Throughput Screen.

[9] S. Wold AR, Wold H, Dunn WJ (1984). The collinearity problem in linear regression. The partial least squares (PLS) approach to generalized inverses. SIAM J Sci Stat Comp.

[10] Jolliffe IT (1982). A note on the use of principal components in regression. Appl Stat.

[11] Malashenko Iu R, Romanovskaia VA, Sokolov IG, Kryshtab TP, Liudvichenko ES (1980). Theoretical evaluation of necessity of carbon dioxide assimilation by microorganisms during growth on various substrates. Ukr Biokhim Zh (1978).

[12] Ajmani S, Jadhav K, Kulkarni SA (2006). Three-dimensional QSAR using the k-nearest neighbor method and its interpretation. J Chem Inf Model.

[13] Molecular Networks GmbH Computerchemie Erlangen, Germany, 1996.

[14] O’Boyle NM, Banck M, James CA, Morley C, Vandermeersch T, Hutchison GR. Open Babel: An open chemical toolbox. J Cheminform. 2011;3:33. doi:10.1186/1758-2946-3-33. 10.1186/1758-2946-3-33 PMC3198950 21982300

[15] VLifeMDS (2010). Molecular Design Suite Pune: VLife Sciences Technologies Pvt Ltd 4.

[16] Gasteiger J, Marsili M (1980). Iterative partial equalization of orbital electronegativity—a rapid access to atomic charges. Tetrahedron.

[17] Derksen S, Keselman H (1992). Backward, forward and stepwise automated subset selection algorithms: Frequency of obtaining authentic and noise variables. Brit J Math Stat Psy.

[18] Kohavi R. A study of cross-validation and bootstrap for accuracy estimation and model selection. International joint Conference on artificial intelligence: Lawrence Erlbaum Associates Ltd 1995, 1137-1145.

[19] Schuurmann G, Ebert RU, Chen J, Wang B, Kuhne R (2008). External validation and prediction employing the predictive squared correlation coefficient test set activity mean vs training set activity mean. J Chem Inf Model.

[20] Rucker C, Rucker G, Meringer M (2007). y-Randomization and its variants in QSPR/QSAR. J Chem Inf Model.

[21] Maestro, Version 9.1, Schrodinger LLC, NY2008.

[22] Kumar V, Kumar S, Rani P (2010). Pharmacophore modeling and 3DQSAR studies on flavonoids as a-glucosidase inhibitors. Der PharmaChemica.

[23] Schoenen FJ, Weiner WS, Baillargeon P, Brown CL, Chase P, Ferguson J, Fernandez-Vega V, Ghosh P, Hodder P, Krise JP (2013). Inhibitors of the Plasmodium falciparum M18 Aspartyl Aminopeptidase.

[24] Cole JC, Nissink JWM, Taylor R (2005). Protein ligand docking and virtual screening with GOLD.

[25] Sivaraman KK, Oellig CA, Huynh K, Atkinson SC, Poreba M, Perugini MA, Trenholme KR, Gardiner DL, Salvesen G, Drag M et al. X-ray crystal structure and specificity of the Plasmodium falciparum malaria aminopeptidase PfM18AAP. J Mol Biol. 422(4):495-507. 10.1016/j.jmb.2012.06.006 22709581

[26] Jones G, Willett P, Glen RC, Leach AR, Taylor R (1997). Development and validation of a genetic algorithm for flexible docking. J Mol Biol.

[27] Wang R, Lai L, Wang S (2002). Further development and validation of empirical scoring functions for structure-based binding affinity prediction. J Comput Aided Mol Des.

