# Supplementary material for: 3D QSAR, pharmacophore and molecular docking studies of known inhibitors and designing of novel inhibitors for M18 aspartyl aminopeptidase of Plasmodium falciparum
Source: BMC Struct Biol. 2016 Aug 17;16:12. doi: 10.1186/s12900-016-0063-7 (PMC4989538; doi:10.1186/s12900-016-0063-7)
Supplement: Additional file 1: — The statistical parameters of 3D QSAR models of known bioactive Inhibitors (AID 743024) dataset of PfM18AAP using PLSR, PCR and kNN-MFA methods. (DOCX 16 kb) [file 12900_2016_63_MOESM1_ESM.docx]

**Additional file 1**

The statistical parameters of 3D QSAR models of known bioactive Inhibitors (AID 743024) dataset of *Pf*M18AAP using PLSR, PCR and kNN-MFA methods.

| **Statistics: PLSR**   \| **Optimum Components** \| 3 \| \| --- \| --- \| \| **n** \| 16 \| \| **Degree_of_freedom** \| 0 \| \| **r^2^** \| 0.8863 \| \| **q^2^** \| 0.6128 \| \| **F_test** \| 31.1820 \| \| **r^2^_se** \| 0.1677 \| \| **q^2^_se** \| 0.3095 \| \| **pred_r^2^** \| 0.6101 \| \| **pred_r^2^se** \| 0.3028 \| | **Statistics: PCR**   \| **Optimum Components** \| 3 \| \| --- \| --- \| \| **n** \| 16 \| \| **Degree_of_freedom** \| 0 \| \| **r^2^** \| 0.8671 \| \| **q^2^** \| 0.4277 \| \| **F_test** \| 26.0922 \| \| **r^2^_se** \| 0.1814 \| \| **q^2^_se** \| 0.3764 \| \| **pred_r^2^** \| 0.6014 \| | **Statistics: kNN-MFA**   \| **k Nearest Neighbour** \| 2 \| \| --- \| --- \| \| **n** \| 16 \| \| **Degree_of_freedom** \| 0 \| \| **q^2^** \| 0.7641 \| \| **q^2^_se** \| 0.2161 \| \| **pred_r^2^** \| 0.0366 \| \| **pred_r^2^se** \| 0.4760 \| |
| --- | --- | --- | --- | --- | --- | --- | --- | --- | --- | --- | --- | --- | --- | --- | --- | --- | --- | --- | --- | --- | --- | --- | --- | --- | --- | --- | --- | --- | --- | --- | --- | --- | --- | --- | --- | --- | --- | --- | --- | --- | --- | --- | --- | --- | --- | --- | --- | --- | --- | --- | --- | --- | --- | --- |
