# Supplementary material for: 3D QSAR, pharmacophore and molecular docking studies of known inhibitors and designing of novel inhibitors for M18 aspartyl aminopeptidase of Plasmodium falciparum
Source: BMC Struct Biol. 2016 Aug 17;16:12. doi: 10.1186/s12900-016-0063-7 (PMC4989538; doi:10.1186/s12900-016-0063-7)
Supplement: Additional file 2: — Comparison between different 3D QSAR models using PLS, PCR and KNN methods for predicting pIC50 values of train set and test set of known bioactive Inhibitors (AID 743024) of PfM18AAP. (DOCX 18 kb) [file 12900_2016_63_MOESM2_ESM.docx]

**Additional file2**

Comparison between different 3D QSAR models using PLS, PCR and KNN methods for predicting pIC50 values of train set and test set of known bioactive Inhibitors (AID 743024) of *Pf*M18AAP.

**Train Set**

| S.  No. | IUPAC Name | Actual pIC50 | Predicted  PLSR | Predicted  PCR | Predicted kNN_MFA |
| --- | --- | --- | --- | --- | --- |
| 1 | 4-[(7-chloroquinolin-4-yl)amino]-2-(diethylaminomethyl)phenol | 6.72 | 6.60 | 6.63 | 6.18 |
| 2 | 4-[2-[(7-chloroquinolin-4-yl)amino]ethyl]benzene-1,2-diol | 6.2 | 6.10 | 6.07 | 6.09 |
| 3 | 7-chloro-N-[2-(3,4-dimethoxyphenyl) ethyl]quinolin-4-amine | 6.18 | 6.28 | 6.40 | 6.11 |
| 4 | 4-(1-quinolin-4-ylpyrrolidin-3-yl)benzene-1,2-diol | 6.03 | 5.88 | 5.80 | 6.19 |
| 5 | N-[2-(4-methoxyphenyl)ethyl]acridin-9-amine | 5.85 | 5.70 | 5.63 | 5.77 |
| 6 | 4-[3-(3,4-dimethoxyphenyl)pyrrolidin-1-yl]quinoline | 5.69 | 6.01 | 5.90 | 5.65 |
| 7 | N-[3-(3,4-dimethoxyphenyl)propyl ]acridin-9-amine | 5.65 | 5.50 | 5.48 | 5.69 |
| 8 | N-[2-(3,4-dimethoxyphenyl)ethyl]acridin-9-amine | 5.61 | 5.62 | 5.62 | 5.26 |
| 9 | 3-[2-(quinolin-4-ylamino)ethyl]benzene-1,2-diol | 5.56 | 5.35 | 5.35 | 5.51 |
| 10 | 4-[3-(acridin-9-ylamino)propyl]benzene-1,2-diol | 5.43 | 5.44 | 5.45 | 5.45 |
| 11 | 7-chloro-N-[2-(3,4-dimethoxyphenyl) ethyl]-N-methylquinolin-4-amine | 5.37 | 5.31 | 5.31 | 5.13 |
| 12 | N-[2-(3,4-dimethoxyphenyl)ethyl ]isoquinolin-4-amine | 5.34 | 5.30 | 5.33 | 5.35 |
| 13 | 4-[2-[methyl(quinolin-4-yl)amino]ethyl]benzene-1,2-diol | 5.31 | 5.47 | 5.46 | 5.34 |
| 14 | 4-(1-quinolin-4-ylpiperidin-3-yl)benzene-1,2-diol | 5.24 | 5.47 | 5.46 | 5.63 |
| 15 | 1-benzyl-N-[2-(3,4-dimethoxyphenyl) ethyl]piperidin-4-amine | 5.161 | 5.22 | 5.32 | 5.23 |
| 16 | 4-[2-(3,4-dihydro-1H-isoquinolin-2-yl)ethyl]benzene-1,2-diol | 5.102 | 5.11 | 5.169142 | 5.26 |

**Test Set**

| S.  No. | IUPAC Name | Actual pIC50 | Predicted  PLSR | Predicted  PCR | Predicted kNN_MFA |
| --- | --- | --- | --- | --- | --- |
| 1 | N-[2-(2-bromo-4,5-dimethoxyphenyl) ethyl]quinolin-4-amine | 6.34 | 6.47 | 6.45 | 6.11 |
| 2 | N'-acridin-9-ylethane-1,2-diamine | 6.3 | 6.13 | 6.27 | 5.33 |
| 3 | 4-[2-(acridin-9-ylamino)ethyl]phenol | 6.15 | 6.20 | 6.24 | 5.73 |
| 4 | N-[(3,4-dimethoxyphenyl)methyl] acridin-9-amine | 5.97 | 6.15 | 6.07 | 5.58 |
| 5 | N-[2-(3,4-dimethoxyphenyl)ethyl]-6-ethoxyquinolin-4-amine | 5.85 | 5.77 | 5.87 | 5.33 |
| 6 | 4-[2-(1,2,3,4-tetrahydroacridin-9-ylamino)ethyl]benzene-1,2-diol | 5.4436 | 5.69 | 5.68 | 5.23 |
| 7 | 4-[2-(quinolin-4-ylamino)ethyl]benzene-1,2-diol | 5.4 | 5.25 | 5.25 | 5.38 |
| 8 | 1-[2-(acridin-9-ylamino)ethyl]-3-phenylmethoxypyridin-4-one | 5.327 | 5.63 | 5.61 | 5.33 |
| 9 | 4-[2-[(8-methylquinolin-4-yl)amino]ethyl]benzene-1,2-diol | 5.327 | 5.15 | 5.25 | 5.33 |
| 10 | 4-[2-[(1-benzylpiperidin-4-yl)amino]ethyl]benzene-1,2-diol | 5.187 | 5.79 | 5.88 | 5.49 |
| 11 | N-[2-(3,4-dimethoxyphenyl)ethyl]-N-methylquinolin-4-amine | 4.92 | 5.42 | 5.41 | 5.66 |
